# Evidence that the delay-period activity of dopamine neurons corresponds to reward uncertainty rather than backpropagating TD errors

**DOI:** 10.1186/1744-9081-1-7

**Published:** 2005-06-15

**Authors:** Christopher D Fiorillo, Philippe N Tobler, Wolfram Schultz

**Affiliations:** 1 Department of Neurobiology, Stanford University, Stanford, CA 94305-5125, USA; 2 Department of Anatomy, University of Cambridge, Cambridge CB2 3DY, UK

We previously demonstrated the presence of delay-period activity in midbrain dopamine neurons, and provided evidence that this activity corresponds to uncertainty about reward. An alternative interpretation of our observations was recently put forth in which it was suggested that the delay-period activity corresponds not to uncertainty but to backpropagating TD prediction errors. Here we present evidence that supports our original proposal but appears inconsistent with the alternative interpretation involving backpropagating errors.

Because the activity of dopamine neurons appears to code reward prediction error, it has been suggested that dopamine neurons may provide a teaching signal in analogy to the prediction error found in temporal difference (TD) models of reinforcement learning. Taking the analogy a step further, it has also been proposed that particular TD models may describe the activity of dopamine neurons [1,2]. More recently, we have reported that dopamine neurons show a gradual increase in activity that occurs between onset of a conditioned stimulus (CS) and reward when the CS is associated with uncertainty about the reward outcome [3]. Niv et al [4] have now suggested how a conventional TD model might account for this observation without reference to uncertainty.

Their explanation relies on the fact that, in certain TD models, prediction errors "backpropagate" in time over consecutive CS presentations. In our experiments, on a particular trial a prediction error occurs immediately after reward onset, which occurs 2 seconds after CS onset. According to the backpropagation model favored by Niv et al, on the next trial in which that same CS is presented, an internally timed "prediction error" would occur at a shorter delay, perhaps at 1.9 seconds after CS onset. On each subsequent trial, the error would occur at a shorter delay until finally it immediately follows the onset of the CS. This model would require that neurons show sudden increases or decreases in activity at long but precisely timed delays after stimulus onset. Although the implementation of such a scheme by real neurons is questionable, it nonetheless might account for the observed delay period activation if one makes the additional assumption that neuronal firing rate has a particular nonlinear relationship to prediction error. For example, Niv et al argue that the difference between 1 and 2 spikes per second has a much greater functional impact in terms of prediction error than the difference between 9 and 10 spikes per second. Thus, adding activity across trials, as we did to generate histograms, would result in the appearance of neuronal activation despite the fact that the average activity at all times (except immediately after CS onset) would correspond to a prediction error of zero. Below we present some of the reasons that we are skeptical of the interpretation of Niv et al.

First, the nonlinear relationship suggested by Niv et al between the firing rate of dopamine neurons and the functional prediction error is opposite to the experimentally observed nonlinear relationship between firing rate and dopamine concentration in mesolimbic target regions. Chergui et al [5] found that there is more extracellular dopamine per impulse at higher firing rates than at lower firing rates.

**Figure 1:**
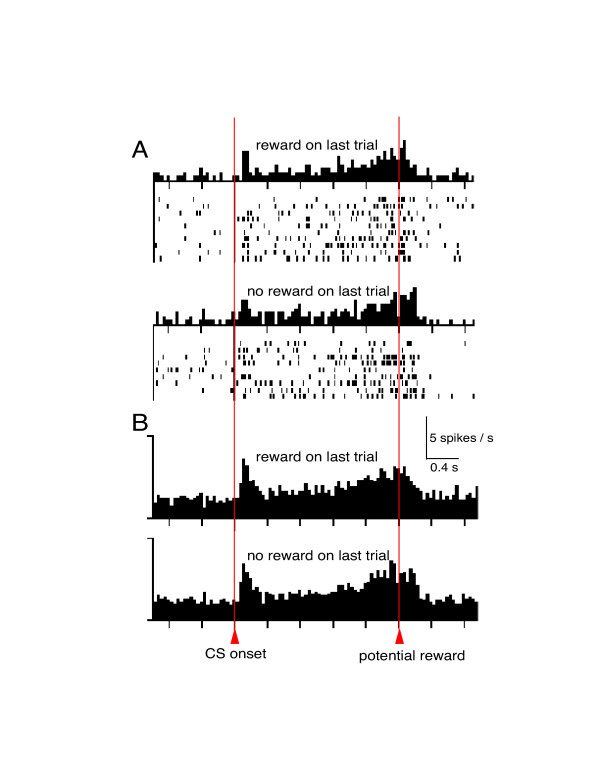
The delay-period activity of dopamine neurons does not appear to depend on the reward outcome of the last presentation (trial) of the same conditioned stimulus (CS). Additional analysis was performed on the data for p = 0.5 from figure 3 of Fiorillo et al [3]. (and reproduced by Niv et al [4] in their figure 1A). Rasters and histograms were generated after segregating trials according to whether the last presentation of the same CS was or was not rewarded. Both rewarded and unrewarded trials are shown. According to the hypothesis of Niv et al, one might expect to see less delay-period activity if the last presentation of the CS was followed by no reward. However, no difference was observed (see statistics in main text). **A. **Activity in a single dopamine neuron. **B. **Average activity in a population of 28 neurons selected for the presence of delay-period activity.

**Figure 2:**
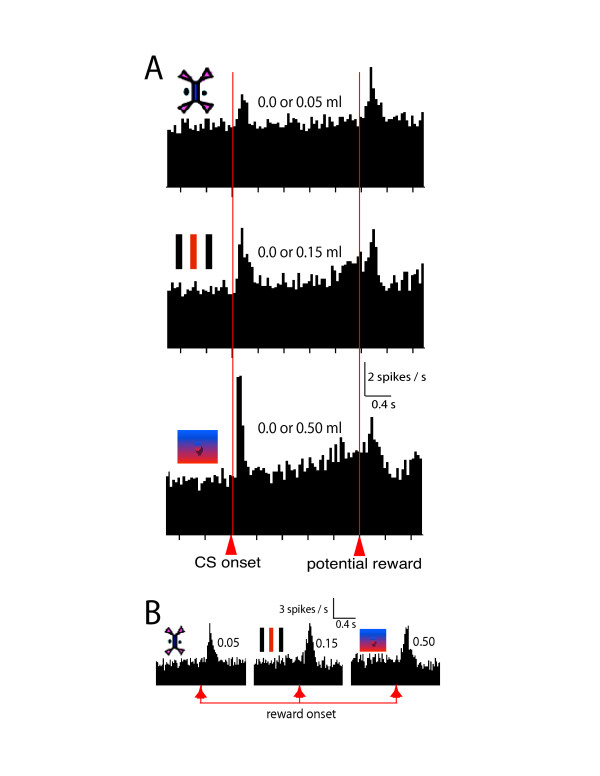
When visual stimuli predict reward at p = 0.5, the delay-period activity of dopamine neurons increases with the discrepancy between potential reward magnitudes, but the phasic response to reward delivery is independent of reward magnitude. **A. **The onset of each of three distinct visual stimuli was followed by either of two potential liquid volumes with equal probability. Histograms represent the average activity of 35 dopamine neurons; these neurons were not selected for the presence of any task-related modulation. Delay-period activity increased with the discrepancy between potential liquid volumes. See figure 4 of Fiorillo et al [3] for a full summary of this data. **B. **Population histograms showing the average response of dopamine neurons to the delivery of reward in the same experiment as illustrated in A (n = 57). Data are from figure 4 of Tobler et al [8], and include data from 22 neurons tested with trace conditioning (as described in [3]) that were not included above in panel A. No differences were observed in the phasic activation to reward in trace versus delay conditioning. The critical observation here is that the delay-period activity varies (from top to bottom) in panel A, but the phasic 'prediction error' response to reward does not (as shown in B). This data is inconsistent with the proposal of Niv et al., according to which the delay-period activity shown in each panel in A should scale with the corresponding phasic prediction error response shown in B. Since the two responses do not scale together, it appears that the delay-period activity cannot be accounted for by the backpropagation of prediction errors.

Second, inspection of the published data appears inconsistent with the model of Niv et al. They suggest that the delay-period activity is an artifact of averaging over trials to generate histograms, and that the sustained increase in activity does not occur in single trials. Contrary to their proposal, there does appears to be strong and sustained activation within single trials, as shown in figure 2 of our original report [3] and in data from another neuron shown here in figure 1A. It is difficult to be certain whether or not activity increases on single trials, in part because Niv et al have not specified precisely what a single-trial increase in delay-period activity should look like, and in part because of the general problem within neuroscience of how to interpret spike trains. Indeed, it would seem that any spike could conceivably represent a backpropagating positive error, and any inter-spike interval could correspond to a negative error. However, if we take a more constrained, conventional approach based on firing rates over tens of milliseconds, then firing rate appears to increase during the delay period on single trials. Similarly, gradual changes in neural activity related to reward expectation are observed in many other types of neuron [[6,7], for example], and are widely believed to represent meaningful increases in activity on single trials rather than artifacts of averaging over trials.

Third, additional analysis of the data (averaged over trials) challenges the interpretation of Niv et al. If the activity during the delay period is due to backpropagating "error" signals that originated in previous trials, then the activity in the last part of the delay period should reflect the reward outcome that followed the last exposure to that same CS. Thus there should be more activity at the end of the delay period if the last trial was rewarded, and less if it was unrewarded. We have analyzed trials in which the CS predicted reward at p = 0.5, and found no dependence of neural activity on the outcome of the preceding trial of the same CS (Fig. 1A,B) (comparing either the last 100 or 500 ms before reward: p > 0.05 in 51 of 54 neurons, Mann-Whitney test; p > 0.4 for the population of 54 neurons, Wilcoxon test). Thus the delay-period activity does not appear to depend on the outcome of the last trial, as suggested by Niv et al.

Fourth, our more recently published results [8] are inconsistent with the model of Niv et al. Each of three conditioned stimuli predicted two potential reward outcomes of equal probability. The discrepancy in liquid volume between the two potential reward outcomes varied according to the CS. The greater the discrepancy, the more pronounced was the sustained, ramp-like increase in neural activity (Fig 2A) [3]. However, the phasic response following reward (or omission of reward) was identical across the three conditions, revealing an adaptation of the prediction error response to the expected discrepancy in reward magnitude (Fig. 2B) [8]. If one were to incorporate these recently published results [8] into the backpropagation TD model of Niv et al, then one would find that since the reward prediction error response at the end of each trial in these experiments is the same, the delay-period activity representing the backpropagating errors would also be the same. However, the data are inconsistent with the model, since the delay period activity increases with the discrepancy between potential reward magnitudes (Fig. 2A) [3]. Our results [8] show that although the phasic activity of dopamine neurons corresponds well to a general definition of reward prediction error, it is inconsistent with the explanation of the delay period activity proposed by Niv et al.

Fifth, it should be noted that the backpropagating prediction error in the model of Niv et al does not reflect an inherent necessity of TD models, but is rather a consequence of the specific temporal stimulus representation chosen. The implementation of different temporal stimulus representations can lead to quite different results. The original TD model [9] and recent versions [10] have used temporal stimulus representations in which the transfer of the neuronal response to the CS is accomplished in a manner that appears more biologically plausible than backpropagation. In TD models utilizing backpropagation, neural signals during the delay period are precisely timed but are without functional consequence, since the sequence of positive and negative errors are self-generated (occurring in the absence of any external events) but are presumed to cancel each other out. This strikes us an odd notion that is neither efficient, nor elegant, nor necessary to the principles of TD learning.

When discrepancies between TD models and responses of dopamine neurons have been noted in the past, such as the absence of depression at the usual time of reward on trials in which reward is delivered earlier than usual [11], TD models have been modified accordingly to better describe the neural activity [10,12,13]. Although TD models have proven very useful, one would not necessarily expect to find the formal structure of any current TD model implemented in the brain. Present TD models exhibit a number of characteristics that appear to be motivated by the need for simplification rather than by any empirical or theoretical constraint. For example, the prediction in TD models is typically equated with expected reward value and ignores the uncertainty in prediction. To illustrate this in terms familiar to people (and perhaps also of relevance to dopamine neurons), a 10% chance of gaining $100 is clearly not equivalent to a 100% chance of gaining $10, yet TD models do not discriminate amongst these two scenarios. TD models have evolved over the years to become more useful and realistic. We believe this process will continue and hope that the study of neurons might be helpful in this regard.
